# Immune Thrombocytopenic Purpura in a Patient With SARS-CoV-2 and Epstein-Barr Virus

**DOI:** 10.7759/cureus.13615

**Published:** 2021-02-28

**Authors:** Arbi Galestanian, Krishna H Suthar, Bernard Karnath

**Affiliations:** 1 Internal Medicine, University of Texas Medical Branch, Galveston, USA

**Keywords:** sars-cov-2, covid 19, immune thrombocytopenic purpura, epstein- barr virus, menorrhagia, thrombocytopenia, peripheral smear

## Abstract

A 35-year-old female was admitted to the hospital for menorrhagia and fatigue. Initial labs revealed that the patient had severe thrombocytopenia and also tested positive for severe acute respiratory syndrome coronavirus 2 (SARS-CoV-2). The main objective in this case is to describe the investigation that eventually led to a diagnosis of idiopathic thrombocytopenic purpura (ITP) in the setting of a SARS-CoV-2 coronavirus disease 2019 (COVID-19) infection and co-infection with Epstein-Barr virus (EBV). The majority of ITP cases are idiopathic and most are diagnosed and managed without hospital admission. Admission and careful management were warranted in this particular case.

Interestingly, however, the patient did not have any respiratory complications associated with COVID-19. She was given 1 unit of platelets and subsequently received intravenous corticosteroids. Platelet counts improved and the patient was discharged with a course of oral prednisone. This case highlights the importance of understanding the differences between primary and secondary ITP.

## Introduction

The patient described in this case presented to the emergency department for evaluation of heavy vaginal bleeding, bruising of skin, and fatigue. Most notable findings on laboratory evaluation were a hemoglobin 10.9 g/dL, a platelet count of 3 per microliter, and serology that was positive for severe acute respiratory syndrome coronavirus 2 (SARS-CoV-2) (both rapid and polymerase chain reaction [PCR]). This patient also tested positive for Epstein-Barr virus (EBV). She was treated with intravenous steroids and received 1 unit of platelets. With respect to the SARS-CoV-2 infection, this patient never showed any signs of respiratory distress and never required supplemental oxygen.

The main purpose of this case report is to demonstrate the importance of thinking about all potential causes for disorders that have a broad differential, such as thrombocytopenia [1]. This case in particular also demonstrated the importance of continuing to investigate for other causes of immune thrombocytopenia even when the most probable likely cause (SARS-CoV-2 infection) has been identified. Multiple cases of idiopathic thrombocytopenic purpura (ITP) in the setting of SARS-CoV-2 infection have been described in the literature during the coronavirus disease 2019 (COVID-19) pandemic. These include case reports (Zulfigar et al., Bomhof et al., and Chen et al.) and systematic reviews (Bhattarjee et al.) [2-5].

As we will see, most cases did not result in any severe complications and responded to treatment with steroids. However, other cases have been published by Bomhof et al., Chen et al., and Martincic et al. in which the patients required administration of intravenous immunoglobulins (IVIG) [3,4,6].

## Case presentation

This patient was a 35-year-old Hispanic female with no past medical history who presented to the emergency department primarily for evaluation of heavy vaginal bleeding, bruises on her skin, and fatigue. Vaginal bleeding had started three days prior to presenting to the hospital. The patient had initially thought that she may be pregnant because the onset of her cycle was late by approximately 15 days. The bleeding improved prior to presenting to the hospital but was still much heavier than her usual periods. At the time of heaviest bleeding, the patient reported changing pads every few hours and occasionally passing clots the size of a quarter. Associated symptoms included fatigue, weakness/malaise, abdominal pain, dizziness, and easy bruising. Review of systems was otherwise negative. 

Further evaluation revealed that she did not have any bleeding disorders, abortions, or previously late/missed periods in the past. As for obstetric history, the patient had two prior pregnancies that had both gone to full term with vaginal delivery. Family history was also unremarkable for any hereditary bleeding disorders, cancers, or other pertinent medical diagnoses. The patient was not taking any medications at time of hospitalization. Surgical history was unremarkable. Regarding social history, she reported cigarette smoking in the past but had quit for several years, occasional alcohol use, and no current or past illicit drug use. Lastly, the patient had been isolating at home with her partner and their two children.

**Figure 1:**
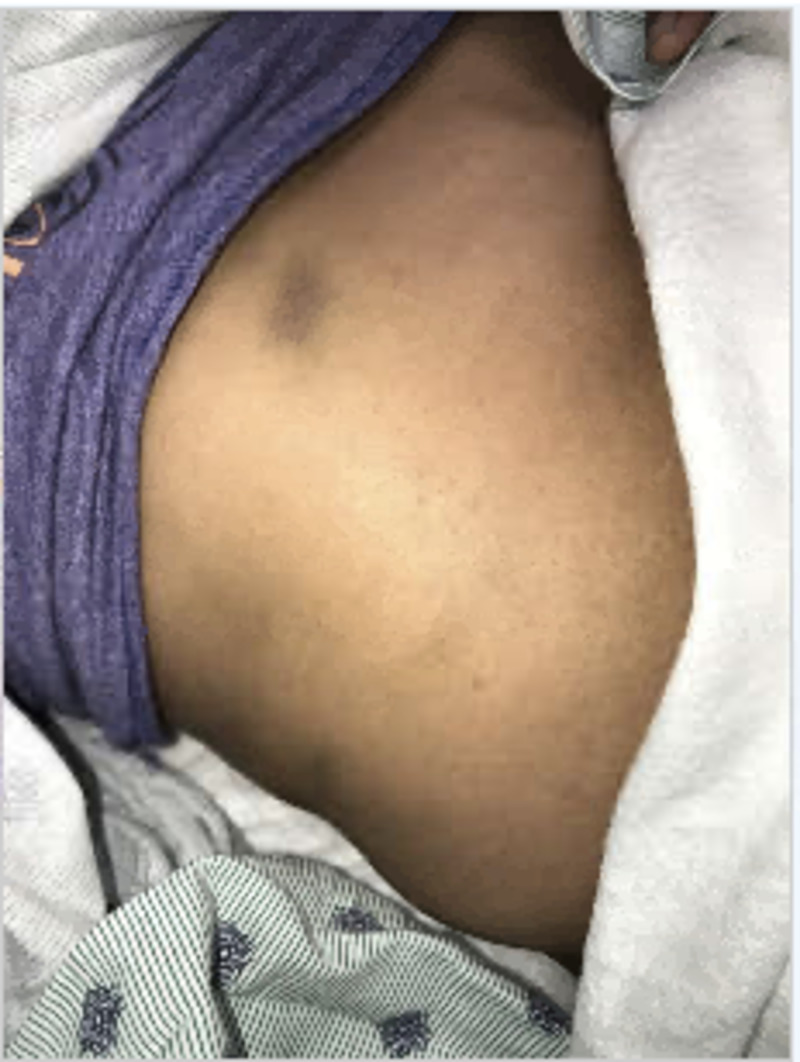
Purpura present on right upper quadrant and less evident on right lateral trunk

**Figure 2:**
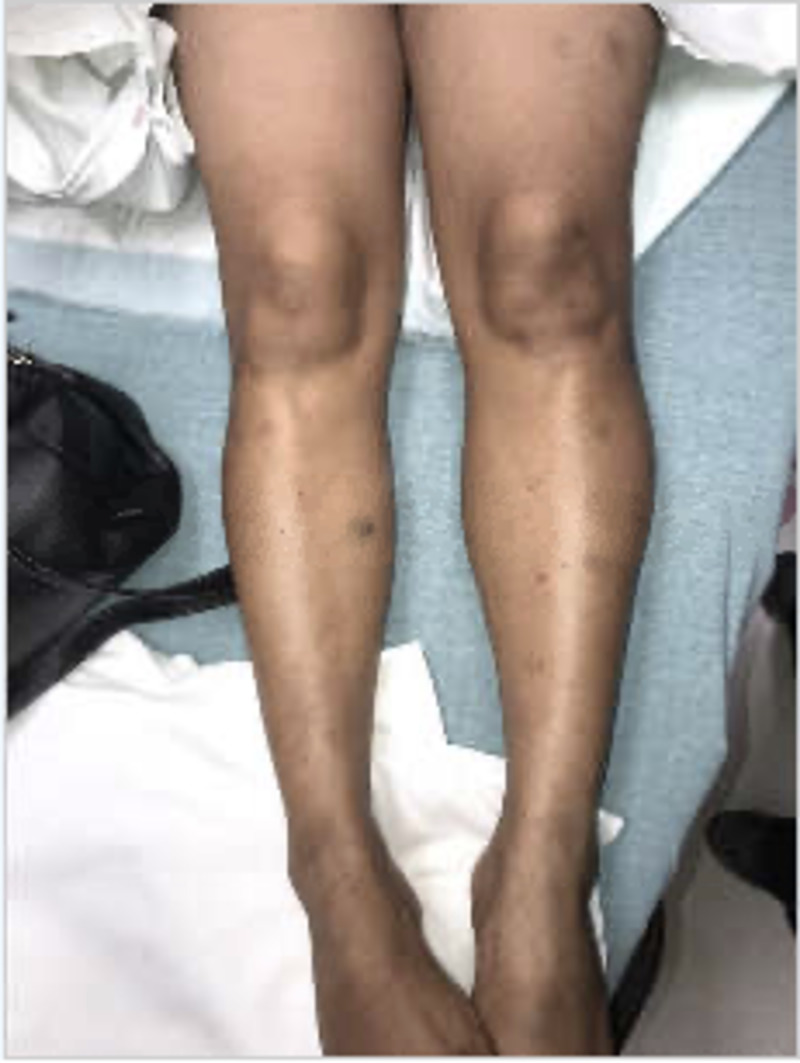
Multiple purpura of varying sizes present on left thigh and bilateral legs

Physical examination at time of presentation revealed the following vital signs: temperature 36.5 Celsius, pulse of 60, respiratory rate 16, pulse oximetry 100% on ambient air, and blood pressure of 110/66. She remained afebrile and never required supplemental oxygen. In general, the patient appeared tired but was not lethargic or somnolent. Examination of the skin revealed multiple non-palpable purpura on her torso and both upper and lower extremities (Figures 1, 2).

## Discussion

The differential diagnosis for low platelets is very broad. Going through all the categories ensures that no possible diagnosis is overlooked and also helps to correctly classify the problem.

As described by Sabatine, thrombocytopenia can be attributed to decreased platelet production, accelerated destruction from consumptive disorders (thrombotic thrombocytopenic purpura [TTP]) or immune-mediated destruction, or sequestration of platelets in conditions causing splenomegaly [1].

The most important step in evaluating thrombocytopenia is looking at the complete blood count (CBC) and peripheral smear. In the case described, the patient also presented with slight anemia that was attributed to menstrual bleeding. The peripheral smear was devoid of spherocytes, schistocytes, or pancytopenia/blasts. In this patient with isolated thrombocytopenia and otherwise unremarkable CBC and peripheral smear, the next major step was to rule out any autoimmune diseases, medication induced, and other infectious etiologies. Other evidence in support of COVID-19-associated ITP included a lower-than-expected response to platelet transfusion and a robust response to first-line treatment. Similar responses were seen in three separate cases of ITP related to COVID-19 infection by Zulfigar et al., Chen et al. and Martincic et al. [2,4,6].

ITP is defined as a rare autoimmune disease characterized by platelet count < 100x109/L, which leads to increased risk for bleeding and bruising. Similar to the case described by Zulfigar et al., the patient described here did not show any signs of bleeding or clotting during hospital admission [2]. Thrombocytopenia has been seen in many cases of COVID-19 and is a risk factor for increased morbidity and mortality. This association is well described in the study written by Chen et al. [4].

Although specific management guidelines are lacking for COVID-19-associated ITP, glucocorticoids are generally the first-line treatment in most cases [2,5]. As seen in the case published by Bomhof et al., some cases of COVID-19 associated ITP, particularly in critically ill patients, fail to respond to high dose corticosteroids necessitating initiation of IVIG [3,4,6].

During the COVID-19 pandemic, it has become widely recognized that the SARS-CoV-2 virus has the capability of generating an extraordinary immune response with devastating multi-systemic consequences. A case describing this 'cytokine storm' was published by Sinha et al. [7]. This phenomenon is explained by immunogenic virus peptides that correspond to human proteins that play an important role in the adaptive immune system. It is becoming clear that the immune response plays a strong role in severe disease presentations of COVID-19 such as COVID-19-associated ITP, as seen in the study written by Martincic et al. [6].

In this particular case, one cannot ignore the fact that the patient had a confirmed co-infection with EBV. Normally, patients with infectious mononucleosis have minor symptoms (fatigue, fever, lymphadenopathy, sore throat lasting up to months). In a study published by Tilden et al., there have been reports of a variety of moderate to severe multi-systemic complications including severe thrombocytopenia [8]. Therefore, we could not rule out the possibility of EBV-associated ITP, or baseline thrombocytopenia due to EBV that became exacerbated by COVID-19 infection [8]. Nevertheless, the overall presentation along with evidence seen in many other cases strongly suggests a causal relationship between SARS-CoV-2 infection and ITP.

## Conclusions

The sequence of events in this case suggested that the patient had secondary ITP due to underlying COVID infection and perhaps co-infection with EBV given the positive EBV early immunoglobulin G (IgG) as mentioned earlier. There was no way to definitively attribute the ITP to either infection; both viruses likely played a role in the patient developing ITP. Despite being infected with COVID-19, the patient never developed any respiratory distress and remained on room air throughout admission. She never developed any spontaneous bleeding or other major sequelae of ITP. This case highlights the importance of investigating all possible secondary causes of ITP. In summary, primary ITP is diagnosed when the evaluation does not reveal other potential etiologies for thrombocytopenia. Secondary ITP is diagnosed in patients with ITP and an underlying associated condition.
